# Deacetylated Konjac Glucomannan with a Slower Hydration Rate Delays Rice Digestion and Weakens Appetite Response

**DOI:** 10.3390/molecules29071681

**Published:** 2024-04-08

**Authors:** Chenfeng Xu, Kaixuan Cheng, Yu Kang, Chao Cheng, Chi Zhang, Longchen Shang

**Affiliations:** 1 College of Biological and Food Engineering, Hubei Minzu University, Enshi 445002, China; 202130358@hbmzu.edu.cn (C.X.); 202230332@hbmzu.edu.cn (K.C.); chengchaolw@126.com (C.C.); zhtzu@163.com (C.Z.); 2 Hubei Key Laboratory of Selenium Resource Research and Biological Application, Hubei Minzu University, Enshi 445002, China; 3 Enshi Tujia and Miao Autonomous Prefecture Academy of Agricultural Sciences, Hubei Minzu University, Enshi 445002, China; es_kangyu@163.com

**Keywords:** konjac glucomannan, deacetylation, simulated digestion, appetite response

## Abstract

The physical characteristics of chyme during gastrointestinal digestion are considered to significantly affect nutrient digestion and absorption (such as glucose diffusion), which has an impact on postprandial satiety. The present study aims to analyze the hydration rate (HR) and rheological properties of deacetylated konjac glucomannan (DKGM) at different degrees and then explore their effects on rice texture, digestive properties, and the subjects’ post-meal appetite. The present results show that, as the deacetylation degree (DD) of KGM increased, the intersection point of the viscoelastic modulus shifted to a high shear rate frequency, and as the swelling time of the DKGM was prolonged, its HR decreased significantly. The results of the in vitro gastrointestinal digestion tests show that the hardness and chewability of the rice in the fast-hydration group (MK1) were remarkably reduced. In contrast, the slow-hydration group (MK5) exhibited an outstanding ability to resist digestion. The kinetics of starch hydrolysis revealed that the HR of the rice in the fast-hydration group was 1.8 times faster than that of the slow-hydration group. Moreover, it was found that the subjects’ appetite after the meal was highly related to the HR of the MK. Their hunger (*p* < 0.001), desire to eat (*p* < 0.001), and prospective food consumption (*p* < 0.001) were significantly inhibited in the slow-hydration group (MK5) compared to the control. This study explored the nutritional effects of the hydration properties derived from the DKGM, which may contribute to modifying the high glycemic index food and provide ideas for the fabrication of food with enhanced satiating capacity.

## 1. Introduction

Dietary fiber (DF) plays a positive regulatory role in the body’s appetite response, glucose and lipid metabolism, intestinal flora, and other aspects [1,2]. Current epidemiological studies have shown that subjects with an adequate intake of DF have a relatively lower incidence of chronic diseases, such as obesity and its associated complications [3]. In addition, the health-promoting effect of DF is considered to be closely related to its physical properties, including viscoelasticity, water holding capacity, and swelling properties in the gastrointestinal tract [4]. Thus, more and more researchers are interested in revealing the nutritive effects originating from its physical properties.

Rice is a staple food nourishing nearly half of the world’s population [5]. Around two billion people in Asia consume rice and its processed products, which comprise approximately two-thirds of their daily calorie intake [6]. Rice contains a high level of starch (75–80%) but is scarce in dietary fiber, which leads to a rapid digestion and absorption rate of the rice starch in the human body [5]. Therefore, the prolonged overconsumption of rice often adversely affects individual health, such as insulin resistance, obesity, diabetes, and other metabolic syndromes [7]. Thus, researchers are becoming interested in utilizing the physicochemical properties of dietary fibers to modulate the digestive properties of starchy foods and thereby improve their nutritional functions.

Konjac glucomannan (KGM) is a natural dietary fiber that can form an extremely viscous sol. This soluble fiber usually hydrates rapidly and significantly reduces the fluidity of the matrix even in its low content, showing strong thickening stability. Meanwhile, those characteristics of the KGM usually lead to the food products becoming too sticky to be manufactured or ingested by people. In addition, following the risk assessment of food additives under the European Commission Regulation (EC) No.257/2010, it is proposed that using konjac in all food categories is limited to a maximum permissible level of 10 g per kilogram [8]. However, the KGM cannot be added to food at a relatively high content at will, greatly limiting its application to daily diet. Therefore, to take advantage of the KGM’s functional properties as much as possible, it is usually modified by removing the acetyl group, limited degradation, and carboxymethylation to expand its application in the food industry [9,10]. Chen et al. [11] studied the properties of the KGM by heterogeneous hygrothermal limiting degradation, and their results demonstrated that it can steeply reduce the apparent viscosity of KGM. This study believes that heterogeneous hygrothermal limited degradation is a practical strategy for KGM degradation with a facile method in the industry. Yin et al. [12] studied the effects of the ultrasonication microstructural properties of the KGM. Their results showed that ultrasonication-degraded KGM had more flexible chain conformation with a lower viscosity and Newtonian flow behavior. The study indicates that ultrasonication is an effective and straightforward method for partially degrading high-viscosity KGM, which can modify the rheological properties and conformation of KGM and further enhance its antioxidant activity.

In the present study, a series of KGM complexes with consistent final viscosity and adjustable swelling characteristics were prepared by modulating the deacetylation degree of KGM. The main physical properties of the mixed konjac flour were characterized, and then the effect of its hydration process on rice’s textural and digestive characteristics was investigated. Based on this, the effect of the konjac flour with different hydration rates on subjective appetites was explored by conducting a consumer test. This research was intended to provide certain ideas for improving the nutritional properties of high glycemic-index foods by applying the KGM, which would be of great significance for developing DF-enhanced rice products and efficiently using konjac resources.

## 2. Results and Discussion

### 2.1. Infrared Spectral Analysis

**Figure 1:**
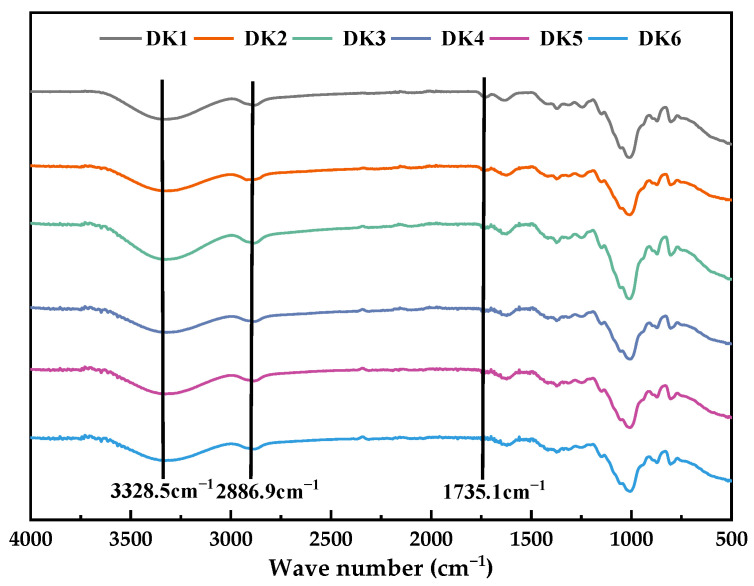
Infrared spectra of KGM with different DD. D1~D6 represent mixed konjac flour with different hydration rates.

The functional groups of the used DKGM molecular chains, were investigated by infrared absorption spectroscopy and are exhibited in Figure 1. The infrared absorption trend of DKGM samples near 3328.5 cm^−1^ and 2886.9 cm^−1^ shows relatively consistent signals, which are the vibration absorption peaks of polysaccharide -OH and C-H, respectively. These results reveal that deacetylation has no effect on the molecular structure of KGM. The stretching vibration peak of C=O at 1735.1cm^−1^, which can be used to indicate the presence of the acetyl group, clearly observes that, as the DD increased, the peak intensity of telescopic vibration decreased, showing that the content of the acetyl group was dropped in the KGM molecular chain [13]. The findings are consistent with the DD values determined in this study and are supported by similar observations made by Ge et al. [14] and Luo et al. [15].

### 2.2. Hydration Process of Konjac Flour

**Figure 2:**
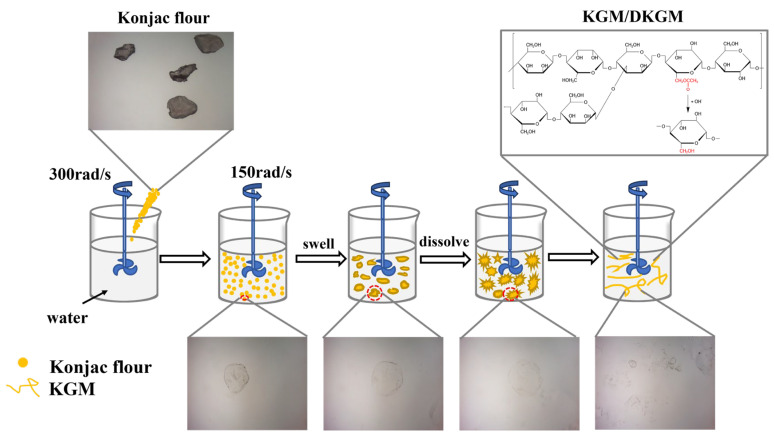
Schematic representation of KGM during swelling in water.

**Figure 3:**
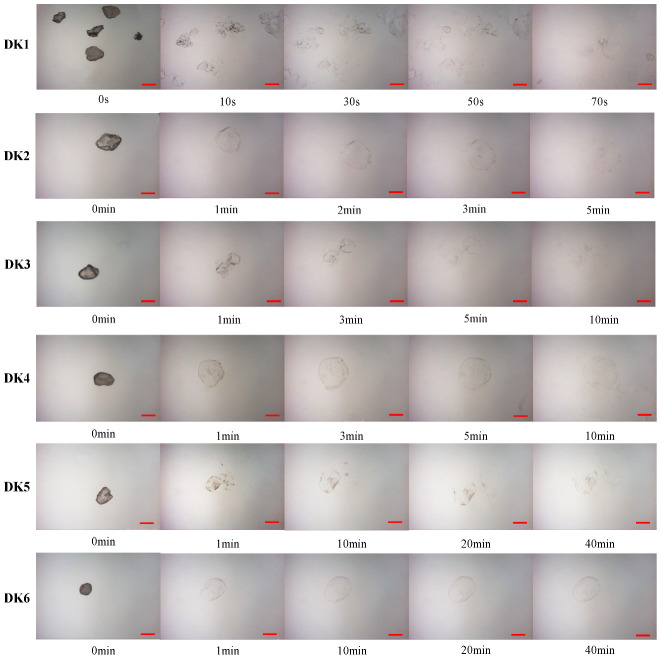
Dynamic swelling process of KGM with different DDs. The red bar in the graph represents 100 μm.

The schematic diagram of konjac flour during dynamic swell is shown in Figure 2. In addition, the hydration of konjac flour particles in simulated gastric fluid (SGF) by optical microscope to visualize the dissolution process is shown in Figure 3. It is evident that the diameter of the konjac flour particles expanded with increasing swelling duration. The native konjac flour reached complete hydration within 70 s, resulting in the formation of insoluble residues, likely composed of cell walls or other insoluble components [16]. However, the dissolution time of DKGM with a DD of 20~65% was substantially extended; the particle morphology was entirely disintegrated for approximately 40 min, while the 95% DKGM remained in a state of partial swelling without complete dissolution. The different degrees of morphological fracture suggest that the deacetylation process may have disrupted the double-helix structure of KGM molecules, leading to their entanglement in a mesh-like configuration. This structural change impedes the easy penetration of water molecules [17].

**Figure 4:**
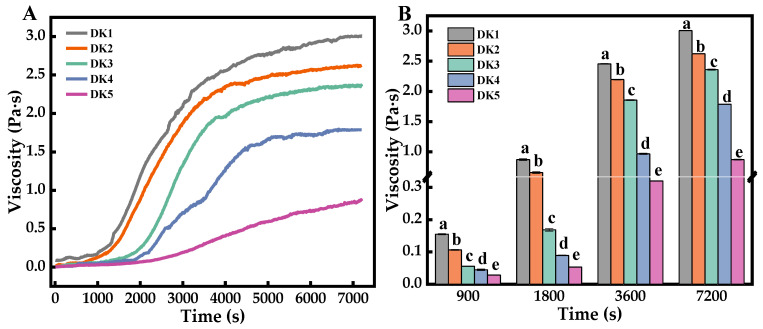
Changes in system viscosity (**A**) and the difference of viscosity (**B**) during hydration of KGM with different degrees of deacetylation. Different letters indicate statistically significant differences between groups (*p* < 0.05).

The viscosity of konjac sol changes during the hydration process with different DDs, as indicated in Figure 4A. The viscosity progressively increases, eventually leveling off at a plateau. This phenomenon may be due to the konjac flour absorbing water to swell, which leads to the dissolving of KGM as a linear molecular chain from the konjac flour. The establishment of hydrogen bonds and molecular dipole interactions between water molecules and KGM molecules contributes to a decrease in fluidity, thereby enhancing the viscosity of the system [18]. Once the konjac flour is fully dissolved, the viscosity stabilizes, reaching a plateau phase. As illustrated in Figure 4B, the viscosity of the KGM sols across different DD groups shows significant differences at various time points (*p* < 0.001). These differences can be linked to the varying acetyl content, which in turn affects the hydrogen-bonding patterns among KGM molecules, leading to distinct rheological behaviors.

### 2.3. Rheology Property of DKGM

**Figure 5:**
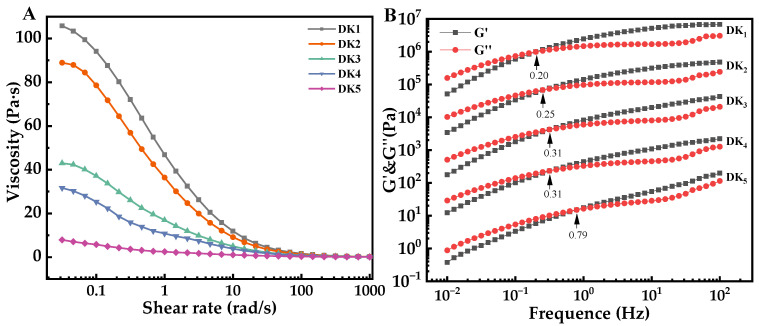
Steady-shear viscosity (**A**) and oscillation frequency sweep (**B**) of KGM sol with different degrees of deacetylation.

The steady flow behavior of the konjac sol (1 wt%) at 25 °C is shown in Figure 5A. The viscosity of the sol decreases with increasing shear rate, demonstrating a pronounced shear-thinning behavior, particularly in samples with a lower DD. Meanwhile, the shear-thinning behavior is much lower for samples with a high DD. This result was mainly due to the external forces exerting gradually larger shear forces on KGM sol, causing the destruction of intramolecular and intermolecular hydrogen bonds of KGM and weakening intermolecular forces, enhancing the fluidity and decreasing the viscosity of the mixed system. Other researchers have also observed similar results [19,20]. To further evaluate the impact of deacetylation on the flow properties of the KGM sol, the Williamson model was employed for a comprehensive assessment and quantitative comparison of key parameters, such as zero-shear viscosity (η_0_) and flow index (n).

The Williamson model expression is as follows:

(1) $$\frac{\eta_{a}}{\eta_{0}}=\frac{1}{1+{\left(\lambda_{w}\overset{\cdot }{\gamma }\right)}^{N}}≅\frac{1}{1+{\left(\lambda_{w}\overset{\cdot }{\gamma }\right)}^{1-n}}$$

where η_0_ is the theoretical zero-shear viscosity (Pa·s), that is, the theoretical apparent viscosity of the fluid at rest; η_a_ is the apparent viscosity (Pa·s) of the sample at a shear rate of (s^−1^); λw (s) is Williamson’s time constant and λw = 1/${\overset{\cdot }{\gamma }}_{c}$, where $\overset{\cdot }{\gamma }$c (s^−1^) is the critical shear rate of the sample, which predicts the starting point of shear thinning in the sample; and n is the flow index, which indicates the flowability of the sample, with higher values indicating superior flow properties. The flow behavior parameters of the samples are listed in Table 1. In the present study, the apparent viscosity of the samples at a shear rate of 50 s^−1^, denoted as η_50_, was considered to be the effective shear rate that can be generated by oral mastication and gastrointestinal peristalsis [21,22]. R^2^ demonstrated the correlation coefficient between the curve and the Williamson model, and all of the R^2^ was greater than 0.99, indicating that the steady flow behavior of the KGM sol could be properly described by this model.

The η_0_ and η_50_ values of the sol progressively decreased as the degree of deacetylation increased. Furthermore, higher η_0_ and η_50_ values have a thicker texture, making them less prone to easy flow during oral ingestion. However, greater η_0_ values are often accompanied by lower critical shear rates, indicating that shear-thinning behavior is more likely to occur in KGM sol with higher η_0_ values when subjected to shearing in the gastrointestinal tract [23]. Consequently, for rapidly hydrating KGM, shear thinning may be the first to occur upon entry into the gastrointestinal tract, which affects the subsequent digestive properties. As a result, this study aimed to modify the physical characteristics of KGM to enable it to fully leverage its nutritional effects on the human body.

The viscoelasticity and molecular chain interactions of KGM can be examined by oscillation frequency scanning. Figure 5B depicts the frequency-dependent behavior of G′ and G″ for KGM sols with different DDs. The dynamic rheological parameters G′ and G″ represent the energy storage modulus and loss modulus, respectively, and their intersection point signifies the onset of polymer chain entanglement. Both G′ and G″ show an increasing trend with frequency, but their growth rates are inconsistent, indicating typical polysaccharide behavior (Figure 5B). At low frequencies, G′ is less than G″, reflecting the fluid-like nature. However, after reaching the crossover point, the upward trend of G′ gradually dominates, reflecting the solid-like nature. The molecular chains of KGM experience prolonged oscillations that unwrap them at lower frequencies. In comparison, the molecular chains cannot unwrap in short oscillations and instead form a temporary spatial network structure at higher frequencies [24]. High-frequency crossovers indicate weaker interactions at the macromolecular level [25]. In the present study, the intersection of the moduli shifts to higher frequencies with increasing DDs, indicating a weakening of molecular chain entanglement in the deacetylated KGM samples. Other researchers also observed this result [20,26].

### 2.4. Apparent Viscosity of MK

**Figure 6:**
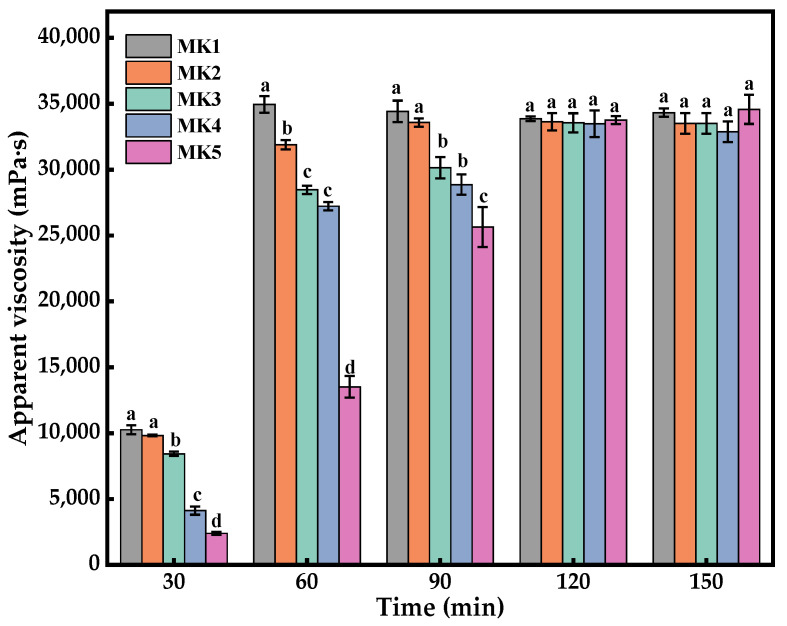
Apparent viscosity of mixed konjac flour. Different letters indicate statistically significant differences between groups (*p* < 0.05).

Five samples of mixed konjac flour (MK) were added to distilled water and mechanically stirred at 150 rpm. The apparent viscosity of the sol was measured by a digital viscometer to monitor the hydration process about every half hour. As depicted in Figure 6, no significant differences in the apparent viscosities of the MK were observed after 2 h (*p* > 0.05). The results demonstrate that a series of MK with consistent final viscosities and substantially adjustable hydration characteristics were successfully prepared. The composite system was subsequently utilized to further investigate the textural and digestive properties of the rice, providing reliable test samples for crowd testing.

### 2.5. Morphological Analysis of Rice after Digestion

**Figure 7:**
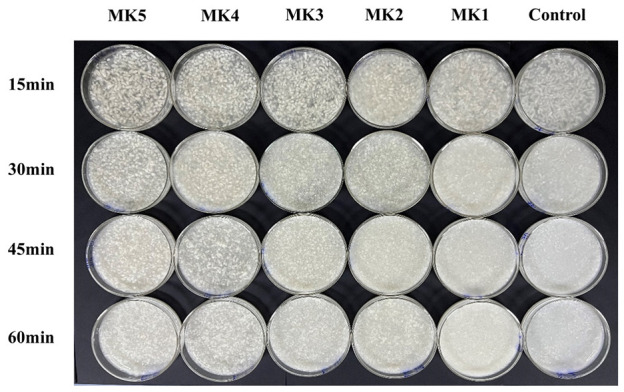
Morphological observation of rice granules after different digestion times.

The rice konjac flour composite system at different simulated gastric digestion stages’ apparent observation is shown in Figure 7. It was discovered that the slow-hydration rate (MK5 and MK4) exhibited significant resistance to digestion when compared with the control group (without konjac flour). This phenomenon revealed that direct contact between α-amylase and pepsin accelerated the disintegration of the cooked rice grain structure. It can be found through this experiment that the physical barrier of KGM sol hinders the chemical digestion of rice during the digestion process.

Newton first described viscosity as a proportional relationship between the flow of a fluid and the force applied to the fluid [27]; in simple terms, when the shear rate is specific, the viscosity of a fluid is proportional to the shear stress, i.e., Newton’s law is expressed as follows:

(2) $$\Sigma \;=\;\eta \overset{\cdot }{\gamma }$$

where σ is the shear stress, η is the shear viscosity, and $\overset{\cdot }{\gamma }$ is the shear rate [28]. As shown in Figure 7, the fast-hydration group showed the fastest rate of rice digestion, and correspondingly, the slow-hydration group was relatively less digestible. In the simulated gastric digestion process, each system’s shear rate ($\overset{\cdot }{\gamma }$) was consistent because the rotational speed of the mechanical stirring paddle was consistent. However, since KGM in the fast-hydration group will rapidly increase the viscosity (η) of the system, according to Newton’s law, the shear stress (σ) received by the system will increase. Although the viscosity increase may hinder the contact between digestive enzymes and rice, which blunt the chemical digestion of rice by digestive enzymes to a certain extent, this effect may not be enough to compensate for the greater degree of rice’s physical digestion caused by the increase in shear stress. In this way, it may lead the rice in the slow-hydration group to maintain a relatively complete appearance and stable texture for a long time during the simulated stomach digestion process. The findings indicate that the viscosity of the system plays a crucial role in the digestion of starchy foods; both excessively high and low viscosities can negatively impact starch hydrolysis and digestion. High viscosity leads to increased shear stress, while low viscosity results in decreased shear resistance and increased sample mobility.

### 2.6. Texture of Digested Rice

Researchers have noticed that the properties of solid food textures have an impact on the rate of gastric emptying, as well as nutrient transportation and absorption in the small intestine [29]. While the majority of studies have concentrated on the intrinsic characteristics of the food, such as particle size, texture, and rheology, less attention has been paid to the effects of food texture on the digestive system, which ultimately affects nutrient digestion and absorption. This study investigated the change in texture properties of food, including hardness, chewiness, springiness, and cohesiveness, during the digestive process, by using MK to create digestive systems with different HR, where hardness was defined as the force required to compress the food to a certain deformation between the tongue and the palate, reflecting the ability of the sample to resist deformation by external forces [30]. Chewiness is the energy required to chew the food to a state where it can be swallowed. Springiness is the rate at which the sample returns to its original state after removing an applied external force. Cohesiveness can be used to characterize the strength of the bonding forces required to form the morphology of a sample [31].

**Figure 8:**
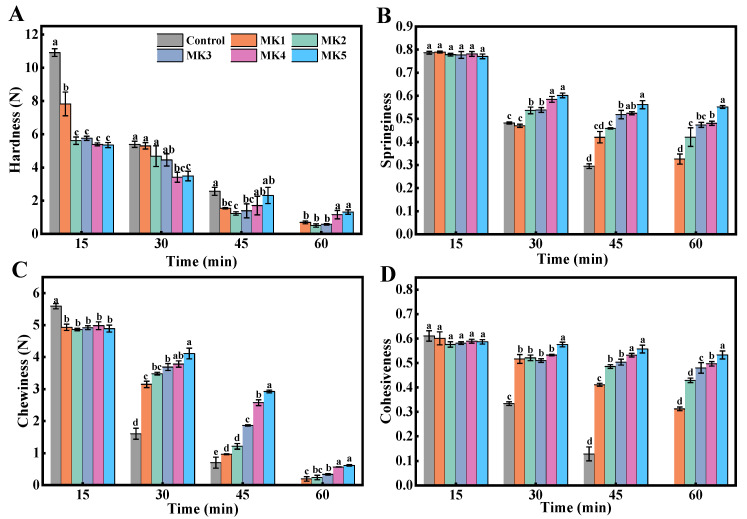
Rice’s texture characteristics of hardness (**A**), springiness (**B**), chewiness (**C**) and cohesiveness (**D**) after the different digestion times. Different letters indicate statistically significant differences between groups (*p* < 0.05).

The hardness of the rice particles decreased with increasing digestion time, as shown in Figure 8A. The slow-hydration groups (MK4 and MK5) had significantly higher hardness than the other groups after 60 min of digestion. This can be attributed to the MK sol adhering to the surface of the rice particles, and due to the fast hydration in a short time exhibiting shear-thinning behavior, the slow-hydration group of MK allowed for continuous hydration and postponed the penetration of digestive enzymes into the rice grains. Undoubtedly, based on the relationship between the disintegration and digestion of solid foods, this will be of significance when modifying high-glycemic-index food and enhancing satiating capacity. As indicated in Figure 8B, a decrease in the elasticity of rice particles occurred during the digestion process. This result may be due the fact that the physical barrier effect of the konjac sol on the cooked rice particles was weakened, caused by the stirring rod’s mechanically destructive force, leading to particles having a higher chance of coming into contact with digestive enzymes and losing their elasticity. However, the digestive system with added MK exhibited greater resistance to digestion when compared with the control group, which was wholly exposed to acid and digestive enzymes. As shown in Figure 8C, the difference in chewability between the groups became more significant as the digestion time increased. This result was primarily due to the corrosive effect of acid and the mechanically destructive force of the stirring rod, which damaged the microstructure of the rice particles to varying degrees [32]. The viscosity of the digestive system increased during rapid hydration, resulting in higher shear stress and friction on the surface of the rice particles. This phenomenon further disrupted the rice microstructure, ultimately leading to reduced chewability.

As shown in Figure 8D, the change in cohesiveness was statistically different between groups. It was hypothesized that the probable reason for this was the formation of interaction forces between the KGM molecules and the starch dissolved in the rice particles. Therefore, the cohesion of the rice particles in the digestive system containing MK was superior to that of the control group. It has been suggested that the textural properties of solid foods can affect the release and transportation of macronutrients and micronutrients in the gastrointestinal tract, which in turn affects satiety [33]. Fang et al. [34] investigated the degree of disintegration of five kinds of cheese with different textural properties in the stomach by in vitro simulated digestion. The results of this study showed that cheeses with lower initial hardness, chewiness, and cohesiveness disintegrated more at the end of gastric digestion, which in turn affected the rate of protein digestion. This study suggests that food digestion kinetics may influence physiological functions such as postprandial protein anabolic responses and increased satiety and muscle protein.

### 2.7. Effects of MK’s Hydration Rate on Glucose Diffusion

After the oral–gastric digestion stage, the rice particles are broken down into starch, protein, and other nutrients, as well as undigested small particles of chyme under the digestive action of α-amylase and pepsin. These then enter the dialysis tubes to simulate intestinal digestion.

**Figure 9:**
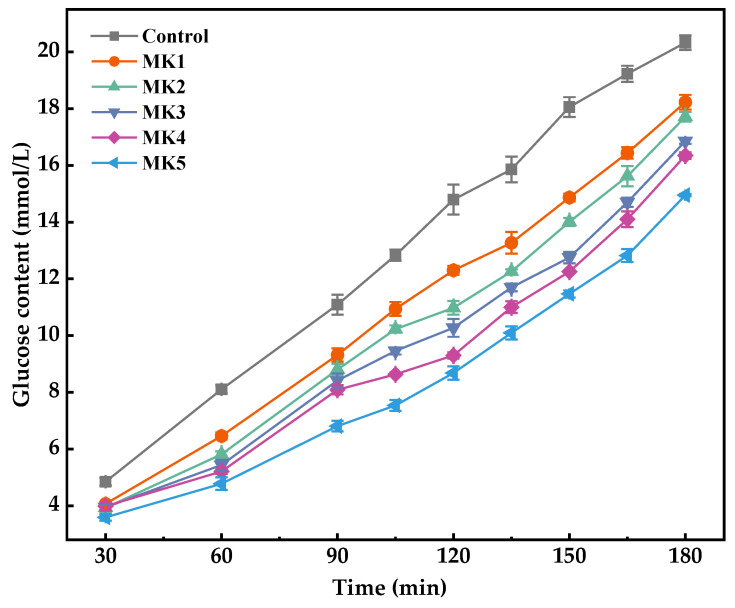
The diffusion concentration of glucose in the digestive juice during the simulated intestinal digestion. Date is expressed as the mean ± standard deviation.

The effect of MK systems on the digestive properties of rice starch was investigated by measuring the glucose content in the dialysate. As depicted in Figure 9, MK had a significant ability to inhibit glucose diffusion when compared with the control group. This inhibitory effect is likely due to the formation of a physical barrier by the MK sol on the rice starch granules, which prevents glycosidases from accessing the starch, thereby hindering its digestion and hydrolysis [35,36]. The slow-hydration rate group (MK5) exhibited the most inhibitory capacity, possibly because it continued to hydrate during the digestion process. Similar conclusions were observed by Guo et al. [37]. Furthermore, non-starch polysaccharides have been identified as α-glucosidase inhibitors capable of binding to the enzyme in competition with starch, thus suppressing starch digestion [38,39].

**Figure 10:**
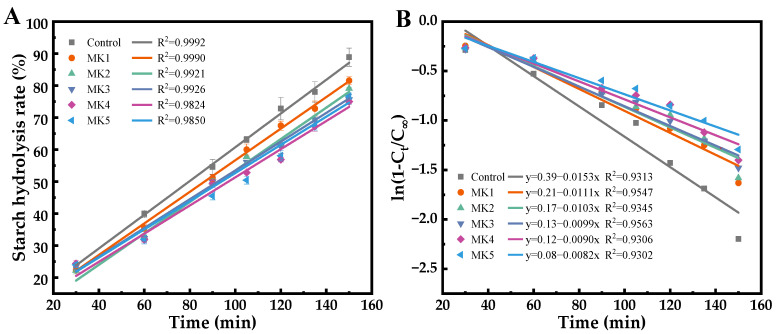
Fitting of rice digestion data to first-order kinetics (**A**) and kinetic constant (**B**). Date is expressed as the mean ± standard deviation.

As illustrated in Figure 10A, the starch content will be obtained when the glucose content has been multiplied by the conversion factor of 0.9. The digestion diagram for starch hydrolysis conforms to the first-order rate equation:

C_t_ = C_∞_(1 − e^−kt^) (3)

where C_t_ is the content of starch digested at moment t (min), C_∞_ is the content of starch digested at the endpoint of the reaction, and k (min^−1^) is the first-order rate coefficient. The value of k can be calculated from the slope of the linear least squares fit of ln (1 − C_t_/C_∞_) to t.

The rice starch digestibility, as depicted in Figure 10A, does not follow the expected curve, possibly due to the presence of small non-free starch particles (<1–2 mm) in the chyme entering the intestine [40] and of glycosidase attached to the chyme’s surface, which hydrolyze the non-free starch. This hydrolysis process is affected by starch particle size, enzyme activity, and digestion rate, so it does not show the typical trend of starch hydrolysis. This work focused on differences in starch digestion between groups. To better explain the effects of different hydration rates on rice starch digestibility, the data were fitted using a first-order kinetic equation (Figure 10B). The ln(1 − C_t_/C_∞_) presents a linear relationship with t. The fitted k value is illustrated in Figure 10B, and the slope k value of the linear curve ranges from 0.0082 to 0.0153. The size of the k value indicates the sensitivity of starch hydrolysis in starch or starchy foods [41], and the greater the k value, the faster the rate of digestion and hydrolysis. It can be seen that the k value of the control group was 0.0153, which was 1.8 times greater than that of the slow-hydration rate (MK5) digestion group, and demonstrated that it could be attributed to the digestive enzymes and mechanical agitation, which may have weakened the binding ability of fast-hydration MK sol to the starch granules. In contrast, the slow-hydration MK sol shows a certain anti-digestion capacity.

### 2.8. Postprandial Subjective Appetite

**Figure 11:**
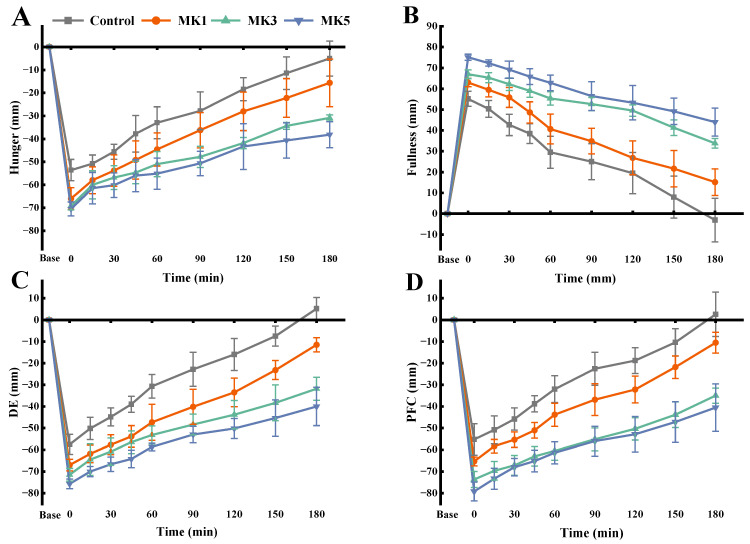
Visual analog scale scores of hunger (**A**), fullness (**B**), DE (**C**), and PFC (**D**) were rated from baseline through 180 min after consumption of the test lunch. Date is expressed as the mean ± standard deviation.

In this study, the dynamic hydration process was delivered to the human digestive tract through the immediate ingestion of a test meal containing MK by the participants, which has an impact on the postprandial subjective appetite changes in hunger, fullness, desire to eat (DE), and prospective food consumption (PFC), as depicted in Figure 11. As the HR of MK decreased, the participants’ perception of hunger within 3 h was weakened (Figure 11A) when compared with the control group. A similar trend can also be observed in the DE (Figure 11C) and PFC (Figure 11D). This phenomenon could also be related to the increased viscosity of the chyme due to the hydration of the konjac flour in the digestion. According to Shang et al. [23], high-viscosity dietary fiber increases the flow resistance of the chyme, making it more likely to resist extrusion from the duodenum and mechanical force applied to the inner wall of the stomach when passing through the pylorus, which in turn delays gastric emptying. There were statistically significant (*p* < 0.05) differences in hunger, DE, and PFC among the groups with the prolongation of digestion time, which may be attributed to the rapidly hydrating konjac flour and reaching its final viscosity within a short time, exhibiting shear-thinning behavior by gastric extrusion and peristalsis. Moreover, the continuous secretion of gastric fluid led to a decrease in the chyme viscosity. The slow-hydration group had better resistance to gastric fluid continuous secretion dilutive behavior, which continued to hydrate during the process.

As shown in Figure 11B, the subjects exhibited prolonged postprandial satiety when they consumed the test meal containing the MK when compared with the control group, and satiety increased as the MK’s HR decreased in the test meal. This result shows that the MK sol provides a physical barrier on the surface of the rice grains, which hinders the process of digestion and thus has an effect on the satiety of the subjects. Furthermore, based on in vitro digestion results, there was a significant difference in the anti-digestion capacity of konjac flour with different hydration levels. Rapidly hydrated konjac flour increases the viscosity of gastric contents in a short time, and the intragastric shear stress gradually increases, resulting in increased friction between the chyme and a gradual decrease in the size of the chyme, which leads to and promotes gastric emptying.

For a further evaluation of the effect of different HR konjac flour on the subjects’ postprandial appetite, the area under the curve (AUC) at 3 h, as depicted in Table 2, was calculated. The statistical results were significantly different regarding the hunger AUC, fullness AUC, DE AUC, and PFC AUC of the subjects when compared with the control group (*p* < 0.05). The fullness, DE, and PFC AUC of the medium and the slow-hydration groups had no statistical difference (*p* > 0.05), but there was a significant difference compared to the fast-hydration rate group (*p* < 0.05). Based on previous results, the ability of the fast-hydration rate group (MK1) to overcome the dilution effect of the digestive fluid showed a decreasing trend with increasing hydration, while the slow-hydration rate group (MK5) was in a state of continuous hydration during the digestion process. Simultaneously, the chyme’s viscosity increased, leading to the increase in shear stress along with stomach peristalsis. Then, the friction between the chyme increased and the grain size decreased, which affected gastric emptying and controlled appetite response. This study underscores the importance of considering the swelling process of dietary fiber in the gastrointestinal tract, particularly for individuals seeking to manage hunger during weight loss. By modulating the hydration rate of konjac flour, it may be possible to enhance satiety and compliance with weight loss regimens, offering a promising strategy for dietary management.

## 3. Materials and Methods

### 3.1. Materials

Konjac flour, KP30, KGM content ≥ 85%, was obtained from Konson Konjac Technology Co., Ltd., Wuhan, China. Both the uncooked and instant rice were purchased from the local supermarket. Glucose assay kit was purchased from Shanghai Yuanye Biotechnology Co., Ltd. (Shanghai, China). Pepsin (P110928), α-amylase (A109181), pancreatin (P110505), and amyloglucosidase (S10017) were purchased from Shanghai Aladdin Biochemistry Technology Co., Ltd. (Shanghai, China). All other chemical reagents were of analytical grade (Sinopharm Chemical Reagent Co., Ltd., Shanghai, China).

### 3.2. Preparation of DKGM

A total of 30 g of konjac flour was added to 150 mL of 40% (*v*/*v*) ethanol that contained a certain mass of Na_2_CO_3_ and placed in a thermostat oscillator at 40 °C to swell for 6 h (150 rpm). After deacetylation, each sample was washed multiple times with 40% ethanol to eliminate excess alkali, and then flushed with absolute ethanol. The samples were subsequently dried at 50 °C until a constant weight was achieved. The DKGM, with its deacetylation degree varying from low to high (coded as DK1, DK2, DK3, DK4, DK5, and DK6, respectively), was prepared by adjusting to the usage of the Na_2_CO_3_ used in the reaction system.

### 3.3. Determination of Deacetylation Degree

The deacetylation degree (DD) of konjac flour is determined according to the method described by Li et al. [42] with slight modification. The 1.00 g sample was added to 50 mL of 40% (*v*/*v*) ethanol and held at 50 °C for 1h. Then, 1 mL of 0.5 mol/L KOH was added in once it was cooled down to room temperature. The mixtures were saponified in a constant temperature oscillator at 40 °C for 48 h. Excess alkali was then titrated with a 0.02 mol/L standard HCl solution. The average value was obtained by calculating the repeated results from three repetitions of the titration process for each sample. The deacetylation degree was calculated using the following formula:

DD (%) = (V2 − V1)/(V0 − V1) ×100%,(4)

The equation shows the hydrochloric acid volume consumed by the blank control (V0), the native konjac flour (V1), and the deacetylated konjac flour (V2) in milliliters (mL). The calculated deacetylation degrees for DK1, DK2, DK3, DK4, DK5, and DK6 were 0%, 20%, 43%, 55%, 65%, and 95%, respectively.

### 3.4. Characterization of DKGM

#### 3.4.1. Infrared Spectroscopy Analysis

A small amount of adequately dried sample to be tested was taken with a medicine spoon and placed on the iD7 Transmission total reflection accessory of the Fourier Infrared Spectrometer (purchased from Thermo Fisher Scientific, Waltham, MA, USA) for infrared scanning, and before the test, the air was used as the background. The wavelength range was set to be 400−4000 cm^−1^, with a resolution of 4 cm^−1^, and 32 scans were performed.

#### 3.4.2. Hydration Process Monitoring

The flour under observation was evenly and smoothly spread on a slide. The microscope was used to observe the morphological changes, swelling, and hydration process of DKGM immediately after the addition of simulated gastric fluid. The dynamic swelling process in the simulated gastric fluid was recorded at regular intervals.

Following the method described by Guo et al. [43] with minor adjustments, the sample was homogeneously dispersed. Then, it was added to the parr plate carrier of the rheometer and ensured that the dispersion system filled the gap between the carrier and parallel steel plates. The test was performed in peak-holding mode with 40 mm parallel steel plates spaced 1 mm apart and with the shear rate set to 50 s^−1^.

#### 3.4.3. Rheological Measurement

A total of 2 g of sample was added to 198 mL of distilled water and stirred at 150 rpm for 1 h to completely dissolve. The sample was characterized using a rheometer. (1) Steady-shear: the fixture was a 40 mm parallel steel plate with a gap of 1 mm, equilibrated for 2 min before measurement, and the shear rate was varied in the range of 0.01~1000 s^−1^, and the viscosity of the system was recorded with the change in shear rate. (2) Frequency scanning: The fixture used for this test consisted of a 40 mm parallel steel plate with a 1 mm gap, maintained at a constant temperature of 25 °C. A strain of 2% was applied and the system was equilibrated for 2 min, the frequency range was set to 0.1–100 Hz, and the dynamic modulus change curve was recorded with frequency.

### 3.5. Digestion of Rice In Vitro

#### 3.5.1. Simulated Digestive Fluid

Simulated salivary fluid (SSF) was prepared, referring to the method described by Marcano et al. [44]. The components were NaCl (0.88 g), KC1 (0.48 g), NaHCO_3_ (5.2 g), α-amylase (final enzyme amount 75 U/mL), and distilled water and they were all configured to form a 1 L oral simulated digestive fluid, and set aside.

Simulated gastric fluid (SGF) was prepared according to the method described by Sanz and Luyten [45]. NaCl (3.097 g), CaCl_2_ (0.111 g), KC1 (1.103 g), Na_2_CO_3_ (0.604 g), and pepsin (final enzyme amount 2000 U/mL) were dissolved; then, the volume was fixed in 1 L distilled water and the pH was adjusted to 2.0 with HCl.

Simulated intestinal fluid (SIF) was prepared according to the method described by Chang et al. [46]. NaCl (5.4 g), KC1 (0.65 g), CaCl_2_ (0.33 g), pancreatin (final enzyme amount 200 U/mL), and glucoamylase (final enzyme amount 180 U/mL) were weighed in turn; then, the volume was fixed to 1 L, and the pH was adjusted to 7.0 with NaHCO_3_ for later use.

#### 3.5.2. Preparation of Mixed Konjac Flour with Different HRs

As listed in Table 3, the consistent final viscosity and different HRs were prepared by modulating the DKGM mass ratio, which was sequentially coded as MK1, MK2, MK3, MK4, and MK5. MK was added to distilled water and stirred at 150 rpm for 2 h to prepare mixed konjac sol with the same final viscosity. The apparent viscosity of the sol was measured by a digital viscometer (NDJ-8S). Each sample was tested three times in parallel, and the average value was considered as the final result.

#### 3.5.3. Texture Measurement of Rice

Following the experimental procedures from the oral digestion stage of Brodkorb et al. [47] with slight modifications, 1.5 g of konjac flour and 28.5 g of rice in different ratios were taken, 30 mL of SSF was added, and the beaker was placed on a temperature-controlled magnetic stirrer with the temperature set at 37 °C and the stirring time set at 2 min.

During the gastric digestion stage, 50 mL of SGF was added to the oral digestion mixture, the beaker was placed on a stirrer, the temperature was adjusted to 37 °C, and the digestion time was set to 2 h. Five grains of rice were collected at 15, 30, 45, and 60 min after the start of digestion, and the texture properties of the rice were determined by a texture tester, with the rice placed radially in the center of the probe. The model P/36R probe with TPA test mode was utilized for the experiment. Pre-test, mid-test, and post-test velocities were set at 2, 1, and 2 mm/s, respectively. The trigger force was set at 5 g, and the data acquisition rate was 200 pps. The deformation was set at 50%, and the hold time was 5 s.

#### 3.5.4. Glucose Diffusion Measurement

The glucose content in the dialysate measurement method was described by Fabek et al. [48]. The gastrically digested chyme was transferred to a dialysis bag with a molecular weight cut-off of 2000 Da, 20 mL of SIF was added to the dialysis bag, and the two ends of the bag were clamped. The bag was then placed in a beaker containing 400 mL of SIF and stirred gently at 75 rpm to simulate intestinal digestion for 180 min. The dialysis bag was manually inverted every 15 min to ensure complete mixing. Samples of 20 μL were taken at 30, 60, 90, 105, 120, 135, 150, 165, and 180 min. According to the operation instructions, glucose concentration in the dialysate was measured using a glucose detection kit. The absorbance was measured at 505 nm using an enzyme-labeling instrument (purchased from Tecan, Männedorf, Switzerland).

### 3.6. Postprandial Satiety Measurement

#### 3.6.1. Preparation of Test Meal

Three samples of KGM complexes with different hydration rates were selected from the five KGM complexes as fast-hydration rate group (MK1), medium-hydration rate group (MK3), and slow-hydration rate group (MK5). The KGM complex was mixed with heated instant rice and stirred thoroughly. The control test meal had the same composition as the three experimental groups, except that it did not contain MK. The four test lunches provided the subjects with a total of 767 Kcal per portion.

#### 3.6.2. Subjects’ Recruitment

A three-factor eating questionnaire (TFEQ) was provided via the Internet to recruit potentially eligible subjects [49]. In addition to the TFEQ section, the main content of the questionnaire consisted of a survey of the subjects’ health status, eating habits, and willingness to participate. Criteria for selecting the subjects were as follows:

- BMI between 18.5 and 23.9 kg/m^2^;
- Age between 18 and 35 years;
- Breakfast regularity with at least 5 days of breakfast per week.

The exclusion criteria were as follows:

- Unstable weight, weight gain, or loss of more than 3 kg in the last 3 months;
- Unhealthy dietary habits, including smoking and alcohol consumption;
- Patients with gastrointestinal disorders.

The study recruited 18 subjects, but 3 withdrew due to scheduling reasons. The remaining 15 eligible subjects (7 males and 8 females) had an average age of 24.3 ± 1.4 years and an average BMI of 21.1 ± 1.8 kg/m^2^. The study was approved by the Medical Ethics Committee of Hubei Minzu University (approval code: 2023011).

#### 3.6.3. Subjective Appetite

The study was conducted as a single-blind crossover trial, with the order in which each subject participated in the 4 randomized trials. Participants were required to visit the conference room of the Food Science Training Center of Hubei Minzu University four times to complete the study, with a minimum of two days between each visit. Subjects were instructed to abstain from consuming beverages containing caffeine for 12 h before the experiment. Additionally, they were not allowed to consume any food or drink after 10:00 p.m. on the day before the experiment. They arrived at the laboratory between 11:00 and 11:20 a.m. and were instructed to remain seated for 10 min before completing a questionnaire to record their baseline subjective appetite. Subsequently, they were provided with a test lunch and instructed to consume it within 20 min. During the eating process, participants were instructed to refrain from using their cell phones or engaging in conversation. The questionnaire was administered immediately after the meal (t = 0 min) and then at regular intervals over the next 3 h (t = 15 min, 45 min, 60 min, 90 min, 120 min, 150 min, and 180 min). After the last questionnaire was completed, the questionnaire forms were collected.

Subjective appetite perception, including hunger, fullness, desire to eat (DE), and prospective food consumption (PFC), was recorded using a visual analog scale (VAS) [50]. The VAS consists of 100 mm straight lines with descriptions of extreme states of opposite appetites written at each end. Participants were instructed to mark a vertical line on the horizontal line to indicate the intensity of their related appetite by the distance between the intersection point and the endpoint. When completing the questionnaire, participants were instructed not to discuss their appetites with each other.

### 3.7. Data Analysis

All experiments were replicated three times. The data were expressed as mean ± standard deviation and statistical analysis was performed using Origin Pro 2021 (OriginLab Corporation, https://www.originlab.com). Data were tested for homogeneity of variance and normality before one-way analysis of variance. The mean was compared using the Tukey test, and statistical significance was considered at *p* < 0.05.

## 4. Conclusions

Deacetylated konjac glucomannan with a lower hydration rate plays an important role in delaying rice digestion and weakening appetite response. In this study, by removing a part of the acetylation group in the glucomannan molecule, we successfully regulated the hydration rate of the konjac flour. The HR of the konjac flour decreased as the DD increased, which usually caused a low hydrolysis rate in the starch and an inhibited release of glucose, showing strong anti-digestive potential in the gastrointestinal digestive stage. In addition, we found that the konjac flour with an appropriate HR would enhance the feeling of fullness, suppress hunger, and reduce DE and PFC, indicating that the swelling process of konjac flour directed in the gastrointestinal tract is a vital control point in designing food with an enhanced satiating capacity. For example, the konjac flour with a specific DD could be packaged as a functional dietary fiber supplement and used in the prefabricated food industry. This research provided both experimental and theoretical support for applying DKGM in high-glycemic-index foods and functional foods.

## Figures and Tables

**Table 1:** Fitting parameters of rheological properties of DKGM sol.

Group	η_0_ (Pa·s)	n	R^2^	η_50_
DK1	104.284	0.1098	0.9996	2.9202
DK2	89.7114	0.1401	0.9994	2.3283
DK3	43.5777	0.2119	0.9989	1.4205
DK4	28.8039	0.2438	0.9964	1.1225
DK5	8.56849	0.4202	0.9953	0.4083

**Table 2:** Area under the curve of participants’ subjective appetite after test lunch (n = 15).

	Hunger	Fullness	DE	PFC
Control	−5363 ± 1101.7 ^a^	4994 ± 1098.4 ^c^	−4875 ± 481.9 ^a^	−5354 ± 1013.5 ^a^
MK1	−7235 ± 1134.4 ^b^	7048 ± 1050.8 ^b^	−7748 ± 893.4 ^b^	−7319 ± 766 ^b^
MK3	−9076 ± 589.9 ^bc^	9860 ± 468 ^a^	−9389 ± 986.8 ^c^	−10,468 ± 769.7 ^c^
MK5	−9780 ± 943.9 ^c^	11,081 ± 800.9 ^a^	−10,523 ± 589.4 ^c^	−10,953 ± 1176.5 ^c^

Different superscript letters (a–c) indicate significant differences in means (*p* < 0.05).

**Table 3:** The components of mixed konjac flour.

Ingredients	MK1 (g)	MK2 (g)	MK3 (g)	MK4 (g)	MK5 (g)
DK1	1.7				
DK2		2.0			
DK3			2.2		
DK4				2.5	
DK5					3.0
DK6	1.3	1.0	0.8	0.5	
Total (g)	3.0	3.0	3.0	3.0	3.0

## Data Availability

Data are contained within the article.
